# A Rare Case of Calcinosis Cutis Presenting to a Tertiary Care Hospital in Rural Maharashtra

**DOI:** 10.7759/cureus.62304

**Published:** 2024-06-13

**Authors:** Joben Samuel, Pankaj Gharde, Dheeraj Surya

**Affiliations:** 1 General Surgery, Datta Meghe Institute of Higher Education and Research, Wardha, IND

**Keywords:** calcified sebaceous cyst, calcinosis, foot swelling, calciphylaxis, dystrophic calcification

## Abstract

Calcinosis cutis is a common ailment of the skin, caused by the accumulation of calcium salts in the subcutaneous regions, though there are rare case reports from rural Maharashtra region. This condition is usually asymptomatic and might manifest as a single growth or several different-sized growths. Its clinical presentation is observed as nodules or plaques without causing injury to underlying tissues. The condition is reported to be a secondary presentation to trauma, malignancies, and connective tissue diseases and has multifactorial underlying etiologies. Recommendation for treatments including both medical and surgical procedures is contingent upon the manifestation and severity of the condition. The final decision can be based on the results obtained from diagnostic modalities like fine needle aspiration and radiological imaging. This is the case of a 35-year-old male with a swelling on his left foot for the past two years without any associated medical history. The patient was managed by surgical incision and had a good recovery.

## Introduction

Calcinosis cutis is a rare skin condition with different underlying causes for its presentation. It has been majorly categorized into five types, dystrophic, metastatic, idiopathic, iatrogenic, and calciphylaxis, amongst which dystrophic calcification has been reported to be the most common [1]. This classification is based on the serum levels of calcium and phosphate [2]. This disease is usually characterized by the deposition of calcium salts in the skin and subcutaneous region due to different reasons such as autoimmune disorders, trauma, infection, or certain types of skin tumors which can trigger dystrophic calcification [3]. There is no standard therapy recommendation for calcinosis cutis. Treatment and management depend on the disease presentation and severity through a multimodal approach involving both medical and surgical interventions [1,4]. This is the case of a 35-year-old male with a major complaint of swelling over his left foot for two years. The patient was confirmed a diagnosis of calcinosis cutis which was managed by the surgical removal of calcinosis deposits. The procedure was uneventful, and the patient did not have any pain during movement and was content on the aesthetic aspect.

## Case presentation

**Figure 1:**
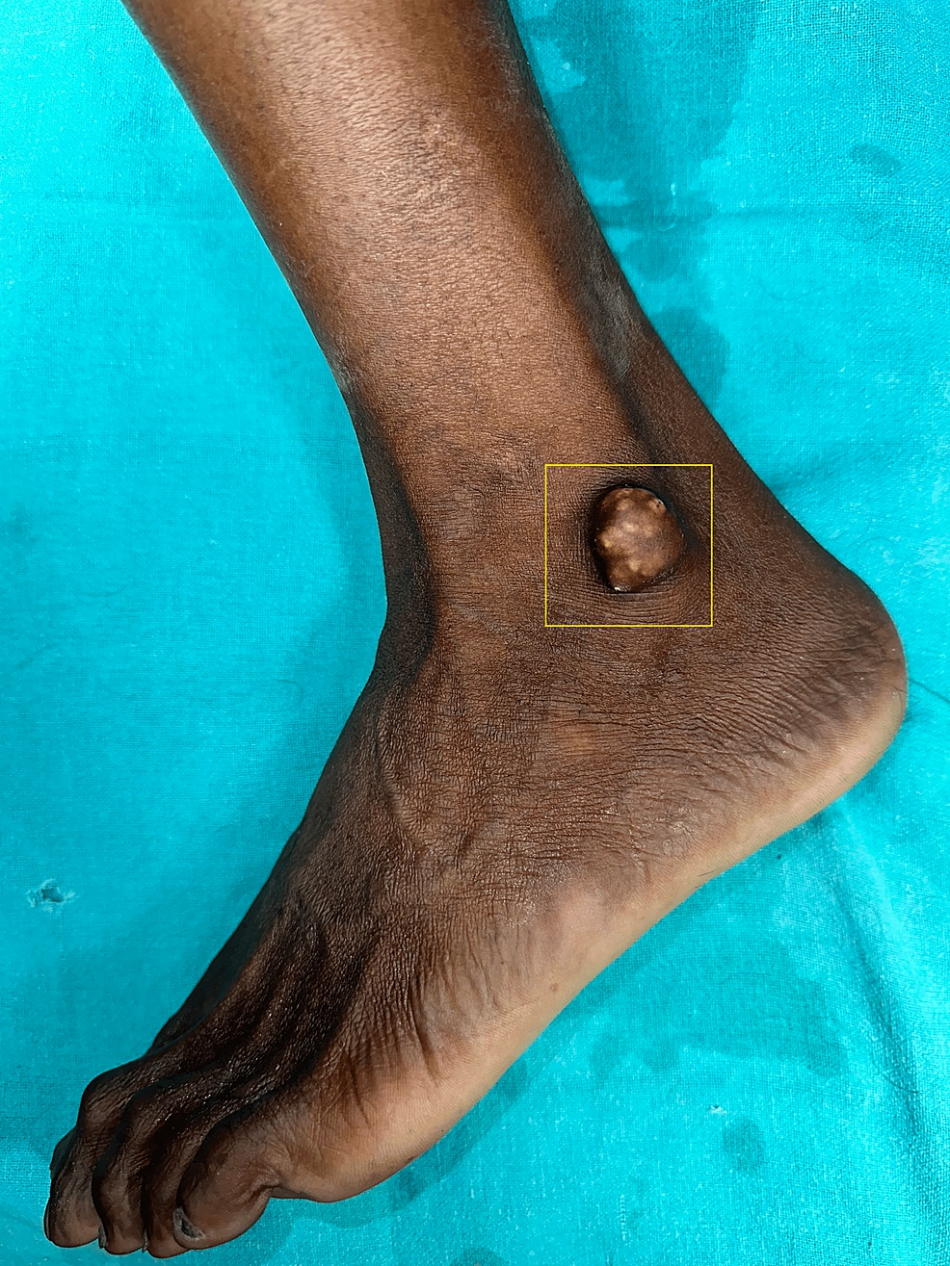
Overview of the swelling of the left foot

A 35-year-old male patient came with a complaint of swelling over the lateral malleolus of the left foot for two years. The patient was thoroughly assessed by a surgeon, which included a detailed medical history review, physical examination, and additional radiological imaging diagnostics which can aid the presence and extent of calcinosis deposits. Clinical examination showed a lesion which was well-defined, firm, and skin color with whitish to yellowish multilobulated nodules 2 × 2 centimeters in size (Figure 1).

**Figure 2:**
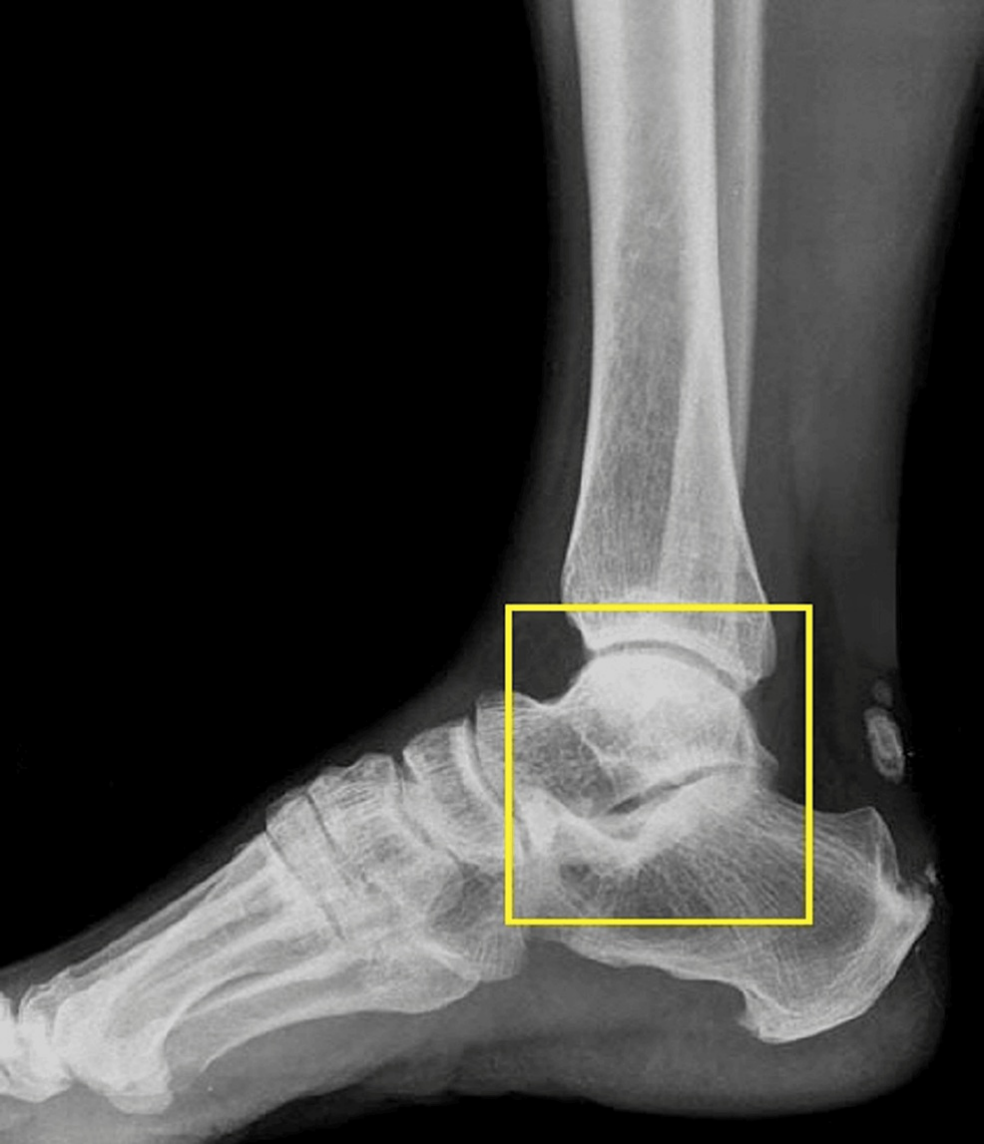
Radiological imaging of the foot

The patient was subjected to ultrasound imaging of the left foot over the lateral malleolus which was suggestive of a calcified epidermoid cyst (Figure 2).

**Figure 3:**
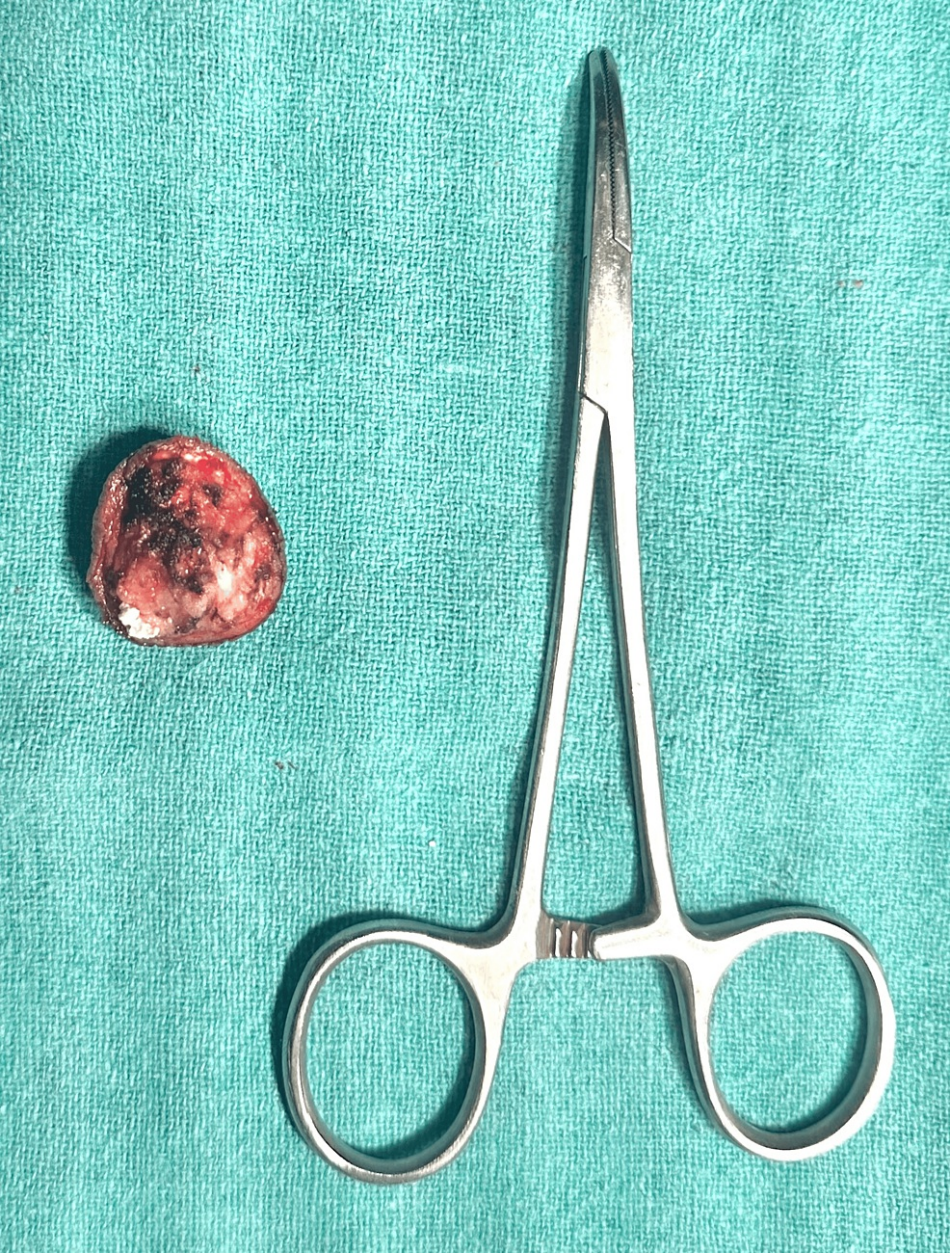
Excised specimen of calcinosis cutis

Fine needle aspiration cytology (FNAC) was done over the lateral malleolus of the left foot which was suggestive of calcinosis cutis. The patient underwent preoperative laboratory tests and medical clearance. The surgical team planned the surgical intervention based on the location, size, and depth of the calcinosis deposits and adjunctive measures to minimize complications. Local plus sedation anesthesia was used in this case. The calcinosis deposits were carefully excised through elliptical incisions to access and remove the calcified tissue while minimizing damage to surrounding structures such as nerves and blood vessels from the affected area of the leg. After the excision of the calcinosis deposits, the surgical incisions were closed using sutures, and the excised specimen was sent for histopathological analysis (Figure 3).

The patient was closely monitored for any signs of complications such as bleeding, infection, or impaired wound healing. Pain management medications were administered as needed, and instructions for wound care and activity restrictions were provided. Post-procedure period was uneventful. Histopathology report was found to be suggestive of calcinosis cutis. The patient is scheduled for regular follow-up visits to assess wound healing, monitor for recurrence of calcinosis, and address any postoperative concerns. Additional treatments such as physical therapy or scar management were recommended to optimize functional and aesthetic outcomes.

## Discussion

Calcinosis cutis is a rare disease with multifactorial etiology such as local tissue damage, caused due to abnormal metabolism, metabolic disorders, side effects, and calcification of blood vessels. It is characterized by the deposition of the salts in the epidermal layers resulting in the physical presentation in some types of calcinosis cutis [5]. These lesions are mostly asymptomatic and can be presented as solitary growth or in multiple numbers with varied sizes [6,7]. Treatment of calcinosis cutis can be challenging. There are different recommendations based on the clinical presentation of the disease. Pharmacological approach uses minocycline, warfarin, bisphosphonates, diltiazem, probenecid, aluminum hydroxide, or ceftriaxone. However, in certain cases, surgical intervention or a combination of both might also be required [3,8]. A retrospective study reported a male-to-female ratio of 6:18 with autoimmune connective tissue disease as the underlying reason in the majority of the patients of the study group. Dystrophic calcinosis cutis was reported as the most prevalent type [1]. There is a similar incidence of calcinosis cutis in all the age groups with a case as young as a two-year-old child [9]. It is considered as a secondary presentation to trauma, tumors, and connective tissue diseases, which is frequently noted as a nodule or plaque of friable nature, of variable size, and without causing any damage to the underlying tissues [3,5]. Radiological imaging studies can be helpful in the diagnosis of calcinosis cutis, but other diagnostic modalities such as fine needle aspiration can also be utilized in the final determination [7,10]. A similar case like this was noted in a 20-year-old male with a solitary subcutaneous nodule near the ankle, where the lateral malleolus was confirmed to have calcinosis cutis on the basis of aspiration cytology which was indicated by the presence of granules of calcium and histiocytes [7].

## Conclusions

Diagnosis and management of calcinosis cutis can be challenging as there are many underlying etiologies. Timely diagnosis can be helpful in good prognostic outcomes. Radiological diagnostic modalities such as X-ray and computed tomography scans are found to be helpful. Though a multidisciplinary approach is recommended based on the presentation and disease severity, surgical excision of the mass is recommended. Postoperative follow-ups are also needed to keep a check on the recurrence of calcinosis, if any. This case highlights the importance of ultrasound imaging and FNAC in concluding the final diagnosis of calcinosis cutis.
